# Ecological role of *Acropora palmata* for its associated species in barrier reefs

**DOI:** 10.1098/rsos.260900

**Published:** 2026-10-28

**Authors:** Gandhi G. Ramírez-Tapia, Anastazia T. Banaszak, Martha A. Gutiérrez-Aguirre, Lorenzo Alvarez-Filip

**Affiliations:** ^1^ Universidad Autónoma del Estado de Quintana Roo (UQROO), Quintana Roo, Cozumel, Mexico; ^2^ Unidad Académica de Sistemas Arrecifales-Puerto Morelos, Instituto de Ciencias del Mar y Limnología, Universidad Nacional Autónoma de México, 77580 Puerto Morelos, Quintana Roo, Mexico

**Keywords:** behaviours, branching coral, coral morphology, habitat use, interactions, reef zones

## Abstract

*Acropora palmata* is a key branching coral in the Caribbean, contributing to reef complexity and providing habitat for diverse species. In recent decades, its abundance and distribution have declined, highlighting the need to better understand its ecological role. Visual surveys along linear transects in the Limones and Picudas reefs (Quintana Roo, Mexico) identified macrofaunal species associated with *A. palmata* and evaluated their interactions to assess habitat use. A total of 81 species were recorded, 34 of which were shared between both reefs. The recorded groups included fish, crustaceans, polychaetes, echinoderms and molluscs. Species composition was similar across both reefs, despite the particular characteristics of each reef. The structure of *A. palmata* was found to generate spatially heterogeneous areas that function as habitat, refuge, feeding sites and territorial spaces at the colony level. Although the colony-specific area influenced species abundance, no effect on size was detected. These findings indicate that, at the colony scale, *A. palmata* provides microhabitats that support diverse assemblages across different reef conditions. This suggests a lack of functional redundancy in the region: *Acropora cervicornis*, its only structural analogue, mainly shelters small-bodied taxa and has become even more depleted, leaving *A. palmata*’s habitat role without a substitute.

## 1. Introduction

Coral reefs exhibit multiple interactions owing to their spatial heterogeneity, which is primarily attributed to the structural complexity provided by various species of scleractinian corals [1–4]. Depending on their morphology and identity, some corals contribute more than others to this complexity [5]. Based on these characteristics, scleractinian corals can be classified into branching, digitate, columnar, encrusting-leafy and massive forms [6]. These morphological variations directly influence the behaviour of resident species, creating spaces that function as physical barriers, such as holes, cracks and branches, which reduce negative interactions like competition and predation and facilitate mutualistic interactions among coexisting species in coral reefs [1,3,7].

In particular, branching corals, such as *Acropora palmata,* are distinguished by their considerable biomass, which, along with their large branching structure, significantly contributes to the structural complexity of the reefs compared with other coral species [8]. For instance, *Acropora cervicornis* shares this branching morphology, but its comparatively smaller colony size limits its contribution to reef structural complexity and the shelter volume it provides [6]. This is generally associated with smaller-bodied organisms, such as juvenile fish [9]. By contrast, the considerably larger size and more robust branching architecture of *A. palmata* gives this species the potential to harbour multiple species of reef invertebrates and fish that benefit from the physical space provided by its structure [10–12]. For this reason and owing to its contribution to other ecological roles, *A. palmata* is considered a key species in the Caribbean [13]. Unlike the reefs of the Indo-Pacific, where ecological redundancy allows various coral species to perform similar functions, in the Caribbean, no other species matches the ability of *A. palmata* to enhance structural complexity and, thereby, its contribution to the ecological balance and biodiversity of the region's reefs [14–17].

*Acropora palmata* predominates in shallow-water zones, forming large, continuous patches, although it is also present in deeper zones generally as isolated and separated coral colonies [13]. Only a few studies have directly examined the effects of *A. palmata* on the presence and behaviour of associated species, such as damselfishes and the crustacean *Domecia acanthophora*, which have a specific habitat association with this coral [4,18]. Other fish, such as grunts, snappers and parrotfish, primarily use the coral as areas of refuge and feeding [8,18,19]. However, the importance of *A. palmata* in modulating the presence and behaviour of a broader range of taxonomic groups that can strictly or facultatively use its carbonate structures is still largely unknown. Moreover, these interactions are likely to be influenced by additional factors, such as wave energy, which varies considerably among different reef zones [19]. For example, the back zone, protected from direct wave impact, tends to have a greater availability of resources compared with areas like the reef crest, where the high energy of the waves creates less favourable conditions for some species [13,19].

Unfortunately, both *A. palmata* and *A. cervicornis* have experienced significant declines in abundance and distribution in recent decades owing to white band disease outbreaks, hurricane impacts and massive bleaching events [8,20,21]. However, recent population assessments across the Caribbean indicate that *A. cervicornis* formations have undergone near-total area reductions and remain critically depleted, whereas *A. palmata* formations have persisted and, in some cases, have maintained stable population sizes [22]. This highlights the need to understand the ecological role of *A. palmata* in hosting other species, which has been documented mainly for one or two taxa [8,20,21], despite its well-established structural importance. This study therefore catalogues the associated species and determines, from their behaviour, how they use the coral and whether size and number of individuals relate to the colony areas available, addressing taxonomically disparate groups simultaneously and testing whether the patterns hold across reef zones and reefs with different habitat quality.

## 2. Material and methods

### 2.1. Study area description

This study was conducted in two reefs of the Puerto Morelos Reef National Park (PNAPM), Quintana Roo, Mexico: Limones Reef (20°59′18.50″ N, 86°47′50.20″ W) located in the northern section of the park and Picudas Reef (20°52′59.80″ N, 86°50′53.60″ W) situated in the central-southern section. Both are barrier reefs with well-defined fore reefs, crests and back reefs (figure 1). These reefs are separated from the coastline by a narrow reef lagoon with a depth of less than 5 metres.

**Figure 1.**
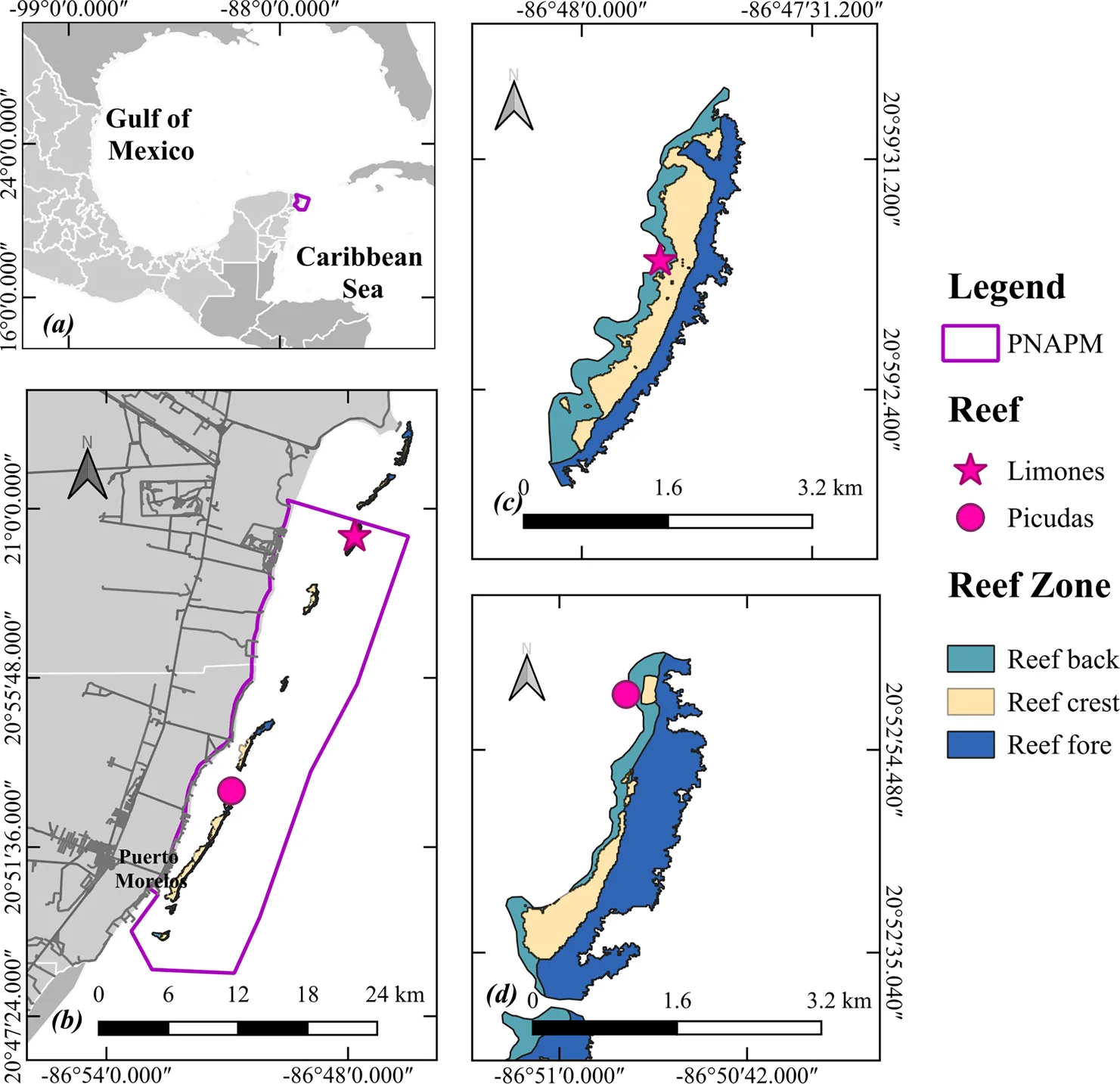
Study area. Geographic location (*a*) and extent (*b*) of the Puerto Morelos Reef National Park. Study sites are marked with a pink star (Limones Reef) and a pink circle (Picudas Reef). The reef zones are shown in detail for Limones Reef (*c*) and Picudas Reef (*d*).

Although several scleractinian corals contribute to reef-building in the Caribbean, the acroporid corals *A. palmata* and *A. cervicornis* have historically been among the principal framework-building species in shallow, high-energy reef zones. Population declines between the two species have nonetheless been markedly asymmetrical across the regions, with *A. cervicornis* suffering comparatively more severe and sustained reductions [22]. At both study reefs, *A. cervicornis* colonies were virtually absent, precluding a balanced comparative assessment; therefore, the study focused exclusively on *A. palmata*.

### 2.2. Data collection

All field activities were conducted under the regulations of the Comisión Nacional de Áreas Naturales Protegidas (CONANP) within the PNAPM. Surveys targeting *A. palmata* were non-invasive and based exclusively on visual census techniques, carried out with prior notification and authorization from park authorities. No organisms were collected, handled or experimentally manipulated.

Belt transects were used to identify species of benthic macrofauna (greater than or equal to 1.5 cm) and ichthyofauna associated exclusively with *A. palmata* corals [23]. Each transect measured 10 m in length and 2 m in width, for a total of 20 m² and was simultaneously surveyed by one observer on each side of the transect to minimize underestimations. Thirty-six transects were established per reef, resulting in a total of 72 transects. The transects were distributed across the three reef zones of each reef. A variable number of transects were assigned to each zone in proportion to the number of *A. palmata* colonies present, resulting in fewer transects in the fore reef zone where colonies were comparatively scarce.

The maximum diameter of each *A. palmata* colony within the transects was measured to calculate the average diameter per transect. The coverage of this coral was estimated using the area formula of a circle, assuming that the colonies were approximately circular in shape. From these data, the average density of individuals present around *A. palmata* per square metre of the sampled area was calculated to standardize the species count associated with this coral.

*In situ* identification of species associated with *A. palmata* was conducted through visual surveys along the previously described transects, using comprehensive Caribbean identification guides [24,25]. Once the transects were established and before beginning the monitoring, 2 minutes were allowed to pass with minimal movement to allow organisms to resume their normal activities [26]. Subsequently, the identification of conspicuous species was conducted for 5 minutes at a distance of 100 cm, followed by closer observations at 50 cm to detect smaller organisms.

This process resulted in a detailed list of species associated with *A. palmata*, identified to the lowest possible taxonomic level. The total number of individuals identified by species and taxonomic group is presented in table S1 in appendix S1 in the supplementary material. Based on the evaluation of the area occupied by the sampled *A. palmata* colonies and the number of associated individuals identified, the density of individuals per square metre was calculated for each zone and at a general reef level. From these data, the average density ± s.d. was estimated.

Categories of use for *A. palmata* were defined based on the behaviour of species relative to the colony. During field surveys, all observed interactions and behaviours of species associated with *A. palmata* colonies were recorded without restriction. For data synthesis and analysis, individual observations were subsequently grouped into a reduced number of habitat-use categories, representing the most frequently observed and ecologically relevant interactions (table 1). Only species that remained within specific zones of the colony were included in this classification, their behaviour and specific interaction areas were recorded. Organisms that merely passed underneath, between or over the coral were neither categorized nor included in the monitoring [14,27].

**Table 1.** Usage categories for species associated with *A. palmata*, summarizing the most common habitat usage behaviours within coral colonies. *Notes:* the four categories are: (i) habitat (H)—species that permanently reside in the colony, (ii) refuge (R)—use of the coral for temporary protection, (iii) territorial behaviour (TB)—species that actively defend their territory on the coral, and (iv) feeding site (FS)—use of the coral for direct or indirect feeding.

groups	habitat (H)	refuge (R)	territorial behaviour (TB)	feeding Site (FS)
fishes	species that permanently reside in coral colonies and maintain a close association with the living coral tissue ^a^	species that use coral colonies as temporary shelter during resting periods or to avoid predators	species that actively establish or defend territories on coral colonies, particularly during reproductive or resting periods	species that visit coral colonies primarily during feeding activities, without permanent residence
crustaceans	species that permanently reside in the structure of the coral colonies^a^	species that use coral structure complexity as temporary protection from predators		species that use coral colonies to feed on associated organisms with the colony
polychaetes	species that bore into the skeleton of the colonies and reside permanently ^a^			species that occur temporarily on coral surfaces while feeding on coral-associated organisms
echinoderms		species that use coral structure for refuge from other predators or during inactive periods		species that occasionally visit the coral structure while feeding on coral-associated organisms

^a^ The species that reside or grow on *A. palmata* were only counted and recorded under the ‘Habitat’ use category.

The definition of categories was determined by a review of the literature, which provided an ecological context for the observed behaviours. The details of the most common habitat use behaviours within coral colonies are presented in table S2 in appendix S1 in the supplementary material.

To analyse the spatial relationships and sizes of species associated with *A. palmata* colonies, five spatial categories within this coral were defined (figure 2). Previous studies have shown that the structure of branching corals reduces interactions, such as competition among resident species [4,8,14,18,27], suggesting that these categories could help better understand the significance of *A. palmata*’s structure for other species.

**Figure 2.**
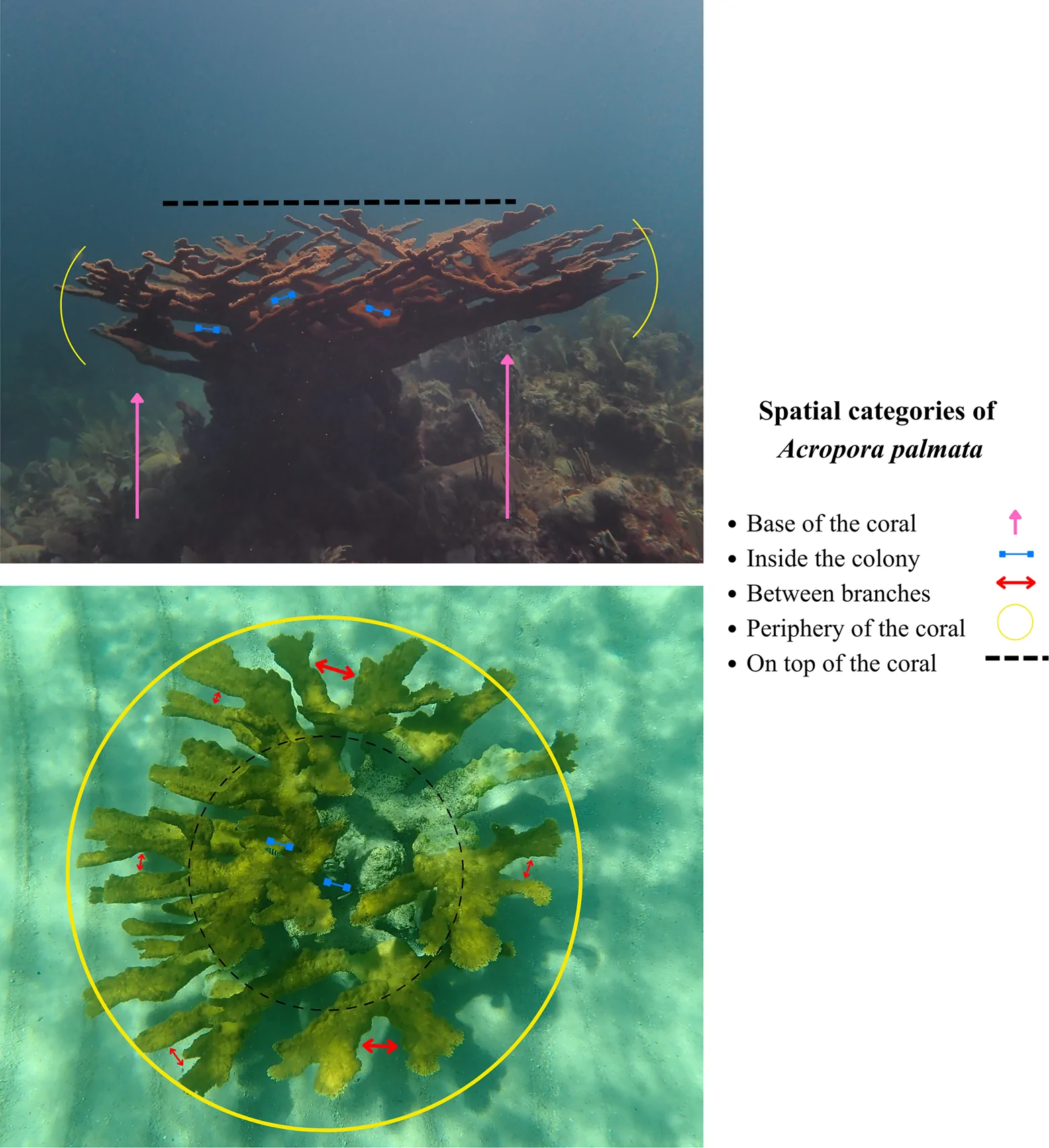
Spatial categories of *A. palmata*. The pink arrow indicates the base of the coral, which includes species found from the substrate up to the first set of main branches of the colony. The blue icon marks the inside of the colony^a^, encompassing species located in the spaces created between the bases of the main branches. The red arrow indicates the area between branches, including species located along and between the main and secondary branches of the colony. The yellow circle highlights the periphery of the coral, including species that remain near colony edges or swim around it as a mechanism of defence or shelter. The black dashed line shows the top of the coral, including species found on the top part of the colony. ^a^Owing to the growth of large biomass of *A. palmata* colonies, they can provide spaces divided by their main branches. Therefore, this category is only applicable to colonies larger than 50 cm.

For descriptive purposes, the most frequently observed behaviour and the most frequently used colony area were summarized independently for each taxonomic family based on the relative frequencies of all observations. These descriptors contextualize the model-based analysis and were not analysed as independent response variables, as both derive from the same set of observations. Similarly, the number of colonies and the area occupied by *A. palmata*, as well as the number of its associated individuals, were summarized per transect.

### 2.3. Statistical analysis

All statistical analysis and figures included in this study were performed and created using R software (version 4.6.1) [28]. To evaluate the efficacy of the sampling effort in this study, accumulation curves of species associated with *A. palmata* were used and differentiated by reef and reef zones. The curves were generated using a random approach with a 95% confidence interval and the non-parametric estimator CHAO 1.

In this study, principal coordinates analysis (PCoA) with chord distance as a measure of dissimilarity [29–31] was employed to visualize the similarity in species composition associated with *A. palmata* across both reefs and their reef zones. When homogeneity in multivariate dispersion was observed, a permutational multivariate analysis of variance (PERMANOVA) analysis with unrestricted permutations was used through the ‘adonis2’ function of the *vegan* package [32] to assess differences between reefs. In cases of heterogeneity in multivariate dispersion, a modified PERMANOVA analysis [33] with unrestricted permutations was performed to evaluate differences between reef zones. Finally, a post hoc test was applied using the ‘pairwise.adonis’ function from the pairwiseAdonis package [34] to identify which reef zones exhibited significant differences in the similarity of species composition associated with *A. palmata*.

To analyse the relationship between habitat use according to the behaviour that species exhibit with respect to *A. palmata*, a generalized linear mixed model (GLMM) [35] was employed. All GLMM analyses were conducted at the transect level. Each observation corresponds to the number of individuals recorded for a given taxonomic group and their behavioural category. The number of individuals was used as the response variable and the behaviour of the species was used as a fixed effect. Owing to the low sample sizes of some species at the species and genus levels, all GLMM were conducted at the taxonomic group level to ensure model stability. Additionally, owing to behavioural differences among different taxonomic groups, these were incorporated into the model as an interaction term with the fixed effect. The GLMM allowed the inclusion of random effects to handle uncontrolled variations from the experimental design [36]. Considering that the species composition is similar among reefs and reef zones, these variables were adjusted as random effects in the model. The inclusion of these effects aided in controlling the variability introduced by external environmental factors. The model was adjusted using the ‘glmer’ function of the lme4 package [37] with a natural logarithmic transformation of the number of individuals to fit them to a lognormal distribution. The model required additional adjustment with a larger number of iterations (100 000), using the ‘bobyqa’ optimizer through the ‘glmerControl’ function of the lme4 package. The validation of the model included diagnostic tests of residuals using the ‘simulateResiduals’ function of the *DHARMa* package [38], including the Kolmogorov–Smirnov test, residual analysis, outlier detection and verification of the residuals' normality, which confirmed the adequacy of the model. The conditional and marginal *R*^2^ values were estimated with the ‘r.squaredGLMM’ function of the *MuMIn* package [39] to quantify the variability explained by the model. Model predictions are reported as estimated average individual counts per transect on the original scale ± standard error. In all the models of this study, the mollusc group was excluded from the analysis owing to its low number of observations (*n* = 4).

To assess how the areas of *A. palmata* colonies influence the number of individuals as well as the size of the associated species, two additional GLMMs were developed, both with a lognormal distribution, following the same transect-level analytical framework described above. The first model included the number of individuals as the response variable, with the areas of the colonies as a fixed effect. The second model considered the size of the species as the response variable, also with the areas of the colonies as a fixed effect. Given the morphological differences among different taxonomic groups, these were included in both models as an interaction of the fixed effect. The models included the reef zones and reef as random effects to control external variability. All adjustment procedures, model validation, estimation of conditional and marginal *R*^2^ and predictions were performed following the same procedures applied in the previous GLMM.

## 3. Results

The accumulation curves of the species associated with *A. palmata* confirmed that the sampling effort was representative of the species diversity associated with *A. palmata* in both Limones and Picudas reefs (supplementary material, appendix S1, figure S1).

*Acropora palmata* colonies in Limones displayed a larger average maximum diameter (210.86 ± 240.86 cm) than those in Picudas (57.19 ± 48.59 cm), with a similar trend observed across all evaluated zones. The Limones reef also had a greater average area occupancy (7.97 ± 17.68 m²) than Picudas (0.44 ± 0.90 m²). In Limones, the crest had the highest occupancy at 22.71 ± 30 m², whereas in Picudas, the back reef had the highest occupancy at 0.75 ± 1.28 m².

A total of 81 species associated with *A. palmata* were identified across both reefs, with 34 species shared between them. Limones hosted 30 unique species, while Picudas had 17 unique species. Limones exhibited a higher density of individuals, with 1004 individuals (2.13 ind m^–2^) compared with 650 individuals (10.68 ind m^–2^) in Picudas. Further analysis of specific zones within each reef revealed that the back reef in Limones had the highest density with 512 individuals (4.25 ind m^–2^), while in Picudas, the fore reef had the highest density with 84 individuals (32.17 ind m^–2^) (supplementary material, appendix S1, table S1). Five main groups of associated organisms were identified in order of abundance: fish, crustaceans, polychaetes, echinoderms and molluscs (figure 3). The species with the highest number of individuals on both reefs were *D. acanthophora* (crustacean, DACA), *Spirobranchus giganteus* (polychaete, SGIG) and *Thalassoma bifasciatum* (fish, TBIF) (figure 3).

**Figure 3.**
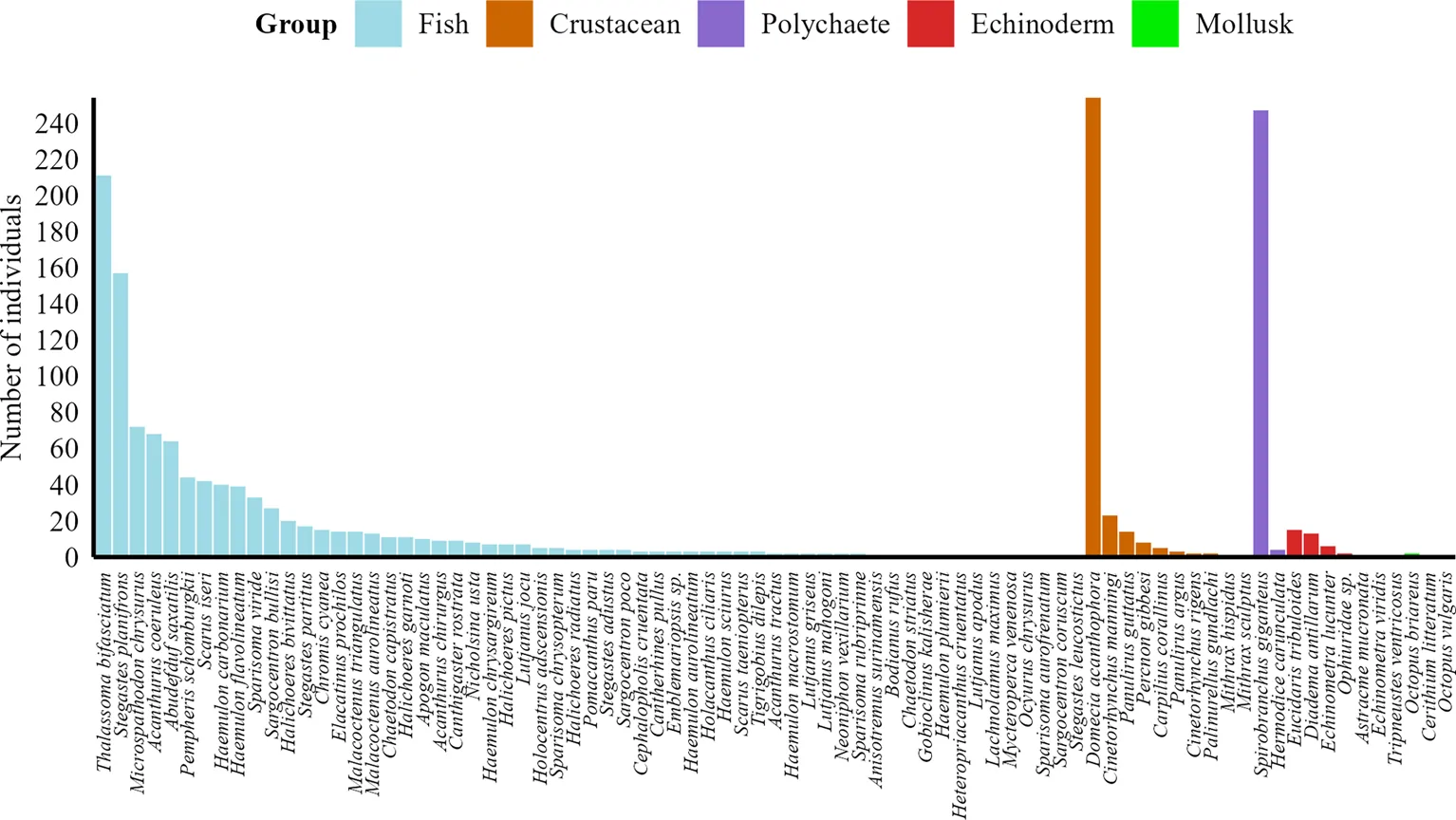
Distribution of the total number of individuals associated with *A. palmata* by taxonomic group. Each bar represents the cumulative number of individuals recorded for each species across all transects identified by a specific code (supplementary material, appendix S1, table S1). Distinctive colours in the graph correspond to different taxonomic groups.

The PCoA for both reefs and their zones revealed that the first two principal components accounted for 22.17 and 18.77% of the total variability, respectively (figure 4). This result suggests significant underlying complexity in the data that these two dimensions do not fully capture, as evidenced by the overlap observed in the ellipses between sites and reef zones (figure 4). This pattern suggests that there are no marked differences in the species composition associated with *A. palmata* across sites and zones.

**Figure 4.**
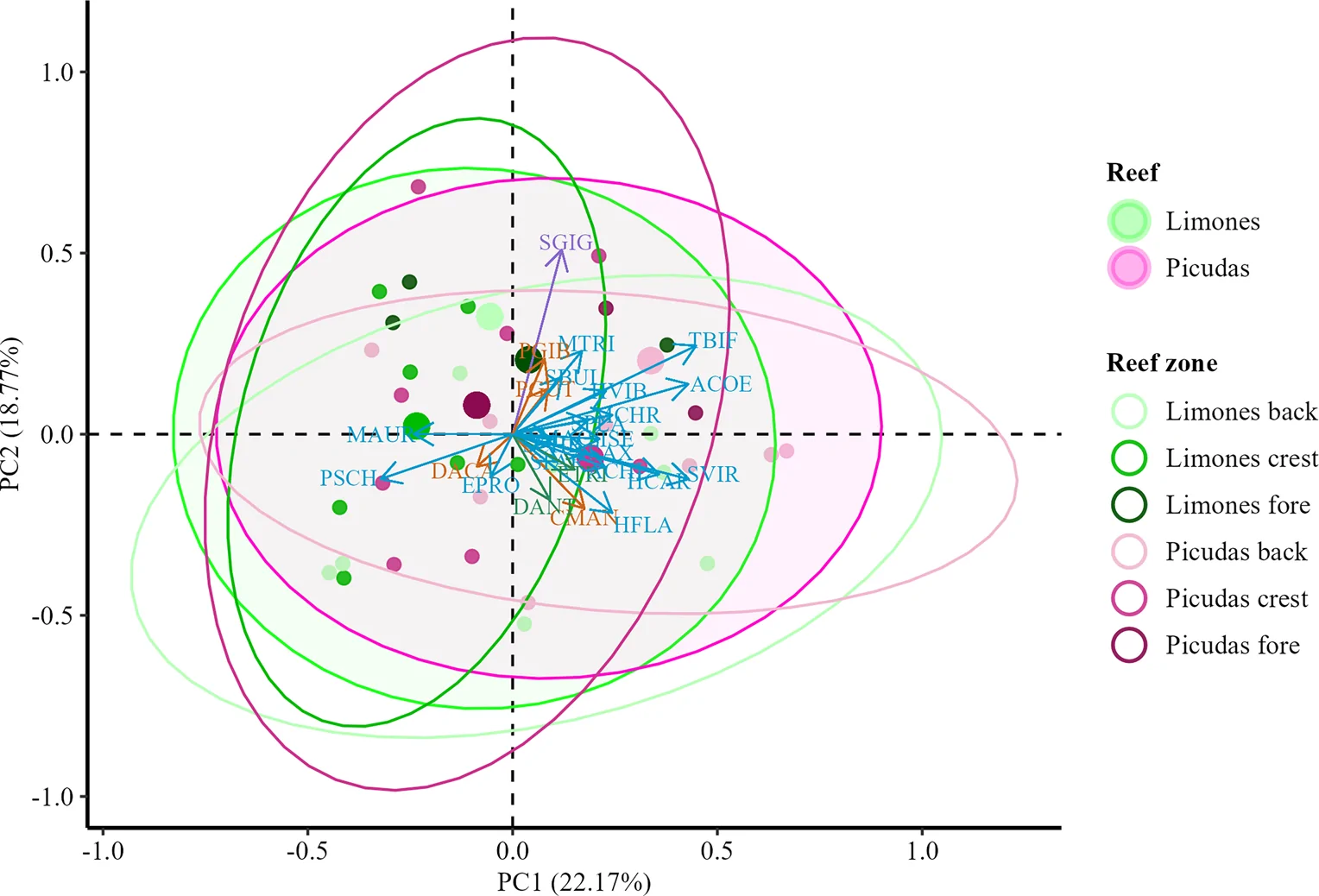
Principal coordinates analysis (PCoA) of relative compositions between the two reefs and their zones. Vectors represent the 27 species associated with *A. palmata*. Larger circles indicate centroids for each reef zone, while smaller circles represent transects. Sample sizes are Limones (*n* = 18), Picudas (*n* = 18), Limones crest (*n* = 7), Limones fore reef (*n* = 3), Limones back reef (*n* = 8), Picudas crest (*n* = 8), Picudas fore reef (*n* = 2) and Picudas back reef (*n* = 8).

Although the hypothesis test indicated a slight trend toward differentiation between sites (*F* = 1.743, *p* = 0.068), this did not reach the conventional threshold for statistical significance. Nonetheless, further statistical analysis identified significant differences in species composition between reef zones (*Pseudo-F* = 1.975, *p* = 0.002). Post hoc tests revealed significant differences between ‘Limones crest’ and ‘Limones back’ (*F* = 2.905, *p* = 0.003), ‘Limones crest’ and ‘Picudas crest’ (*F* = 5.987, *p* = 0.001) and ‘Limones crest’ and ‘Picudas back’ (*F* = 5.255, *p* = 0.001). These results suggest that ‘Limones crest’ contributes uniquely to this variability, potentially owing to the density of individuals in this zone. Therefore, while ‘Limones crest’ exhibits distinct characteristics, the overall evidence suggests that species composition is generally similar across sites and zones.

Furthermore, table S3 in appendix S1 in the supplementary material provides a summary of the most frequently observed behaviours and colony areas by taxonomic family, offering an overview of the distribution of uses by species associated with *A. palmata*.

The result of the GLMM to assess the behaviour of species associated with *A. palmata* showed that, in the random effects, the reef zones did not have a significant effect on the variability in the number of individuals of these species (supplementary material, appendix S1, table S5). The site effect also showed a non-significant influence (supplementary material, appendix S1, table S5) on the number of individuals of these species. The absence of significant effects on these variables suggests that the behaviours of the species are consistent across the different reef zones and sites studied. However, the addition of these effects allowed for a better fit of the model for estimating the fixed effects (supplementary material, appendix S1, tables S5 and S6).

Regarding the fixed effects, a significant relationship was identified in the number of individuals according to their behaviour. However, the group did not show a significant effect on the number of these individuals, although a decrease compared with the intercept was recorded. In the interaction of the fixed effects, it was found that the habitat used by the fish group significantly influenced the number of these (estimate = –1.05, s.e. = 0.29, *t* = –3.65, *p* = 0.001), showing a notably lower value than that of the intercept (supplementary material, appendix S1, table S5).

The predictions generated from this model demonstrated how the expected number of individuals in colonies of *A. palmata* varied depending on their behaviour and group. It was observed that the groups of crustaceans and echinoderms recorded the highest values in terms of habitat-use behaviour (4.697 ± 0.6179 and 5.581 ± 0.7239, respectively). Additionally, refuge and feeding behaviours showed a similar number of expected individuals among the different taxonomic groups. In terms of territorial behaviour, fish and polychaetes presented lower and more uniform values compared with other uses (figure 5).

**Figure 5.**
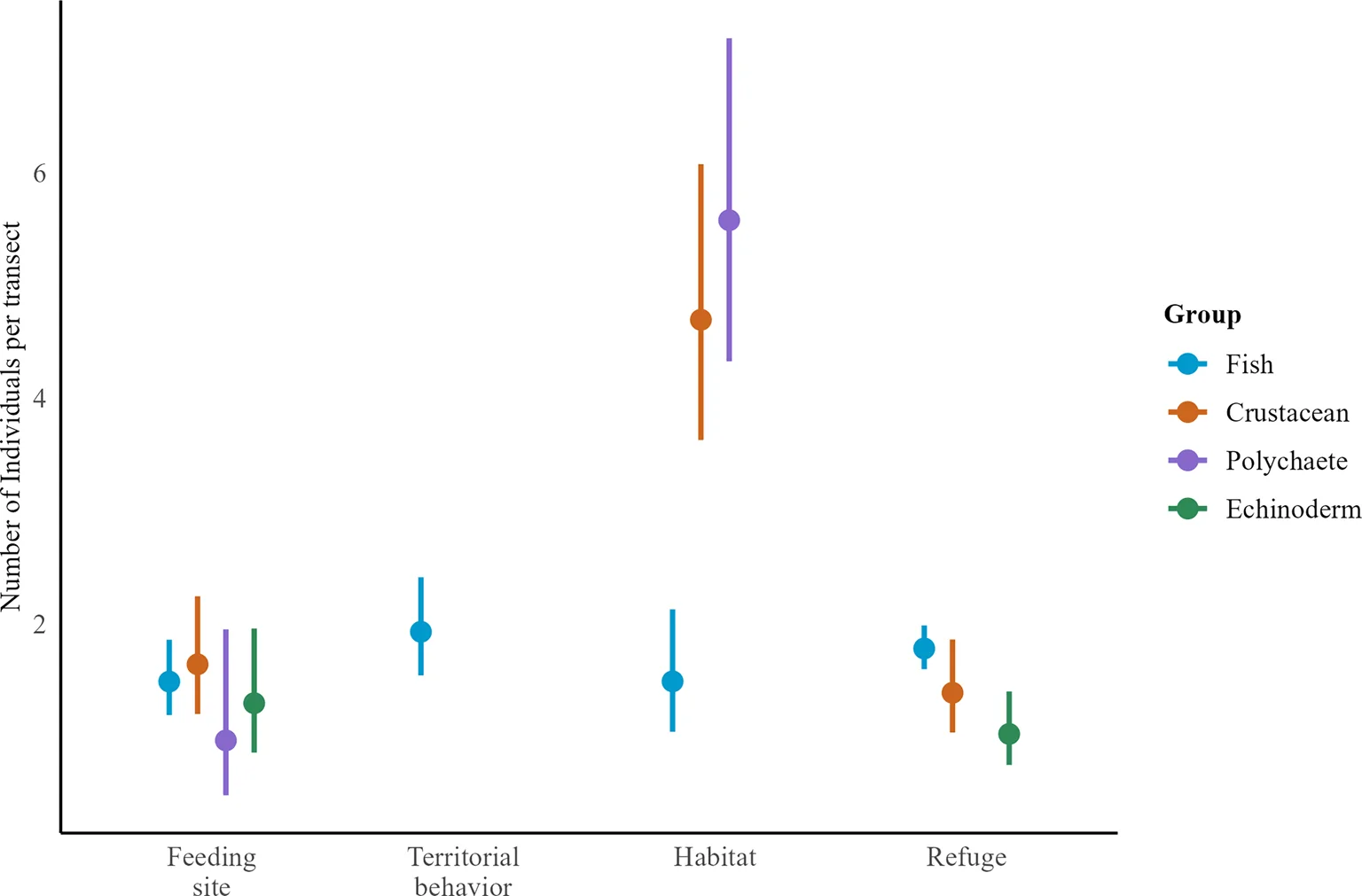
Distribution of behaviours of species associated with *A. palmata*. The points represent the estimated average number of individuals per transect on the original scale. The error bars represent the 95% confidence intervals for each estimated average.

The data on the number and area of *A. palmata* colonies and the number of associated individuals per transect are summarized (supplementary material, appendix S1, table S4). The GLMMs applied to assess the effect of *A. palmata* areas on the number of individuals and sizes of species associated with this coral showed similarities in the random effects. In both models, the reefs and their zones did not have a significant effect on the number of individuals or sizes of these species (supplementary material, appendix S1, tables S7 and S9). However, the inclusion of these effects was crucial for the proper fitting of the models, as it allowed for consideration of the variability associated with these factors and improved the precision of the fixed effects estimates (supplementary material, appendix S1, tables S8 and S10).

In general, the first model showed that the fixed effects and their interactions had a significant effect on the number of individuals associated with *A. palmata*. For the second model, the influence on size was primarily attributed to the colony area, except in the ‘inside’ area, where no significant effect was observed (estimate = 0.277, s.e. = 0.568, *t* = 0.488, *p* = 0.626; supplementary material, appendix S1, table S9).

According to the predictions of the first model, a greater number of individuals were observed in the upper areas of the colony (i.e. ‘periphery’ and ‘on top’) compared with the lower areas (i.e. ‘base’ and ‘inside’), except in the case of echinoderms (figure 6*a*). By contrast, the predictions of the second model indicate that, especially for crustaceans compared with fish, individuals in the upper areas of the colony tend to be smaller in size than those in the lower areas. Additionally, echinoderms did not have a clear distribution pattern relating to their size across different areas of the colony (figure 6*b*). These observations suggest that the various groups might have adapted to occupy specific areas of the colony, possibly to reduce disadvantageous interactions and thus maintain a better ecological balance within the colony.

**Figure 6.**
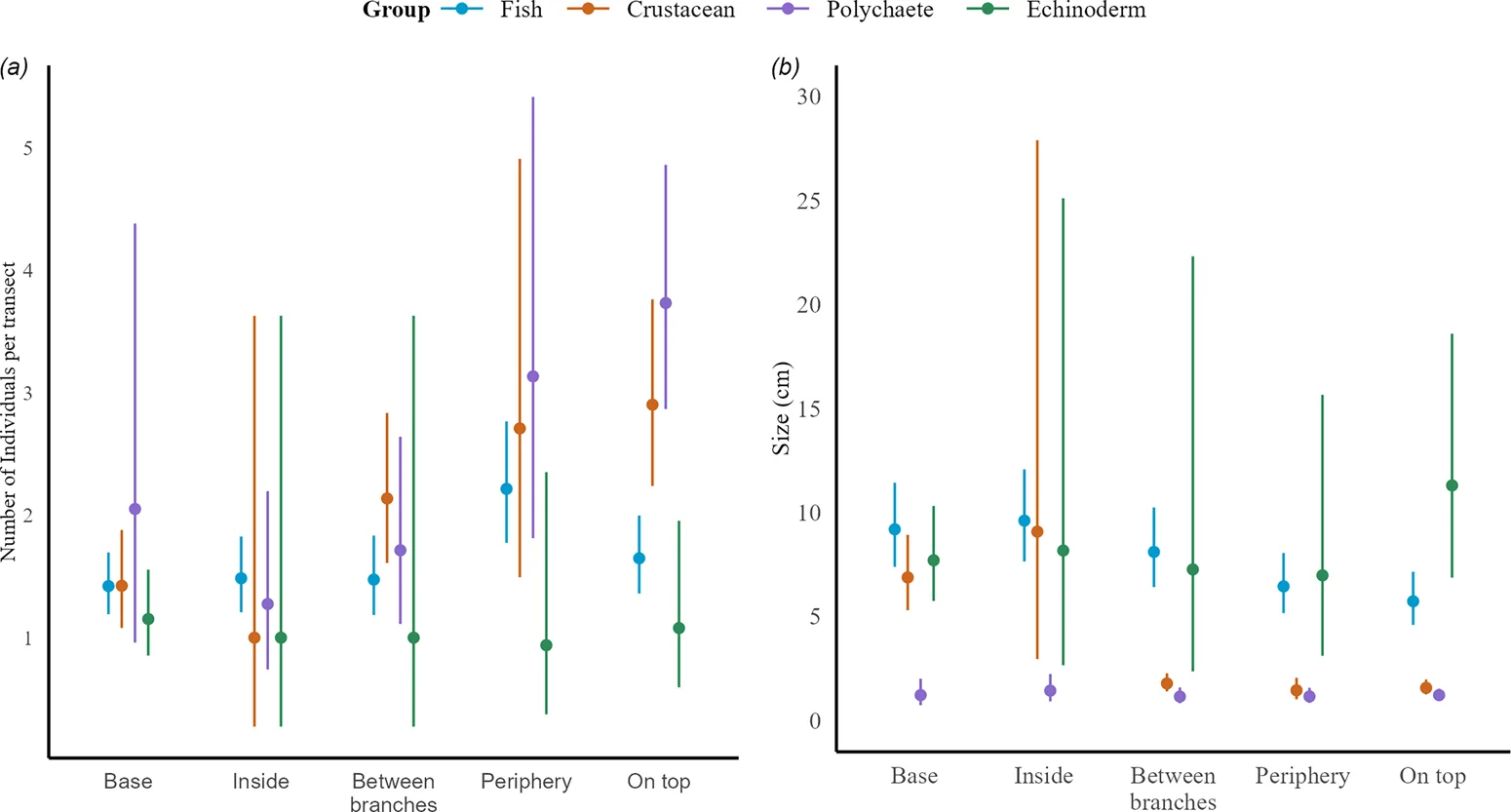
Distribution of the number of individuals and sizes of species associated with different areas of *A. palmata* on the original scale. Estimated number of individuals per transect (*a*) and estimated average sizes of individuals in cm (*b*), predicted by the GLMM at the transect level. The points represent the estimated marginal mean and the error bars indicate the 95% confidence intervals.

## 4. Discussion

The structure of *A. palmata* is proposed in this study as a key component in shaping reef space, as it supports a diverse community of associated organisms represented by a total of 81 species. Beyond the number of species recorded, the similarity in community composition among reefs and across reef zones suggests that the areas formed by *A. palmata* function as available habitat and refuge spaces. Moreover, colony morphology provides distinct areas within its structure, each with different spatial characteristics, allowing for a differentiated distribution of taxa within the colony.

The presence and morphology of *A. palmata* are key elements for the organization of biological communities in reefs with different habitat quality, as observed in the present study. Picudas has been classified as a degraded reef [40], characterized by deteriorated structural and functional conditions [41], whereas previous studies at Limones Reef have documented more favourable conditions and higher habitat quality [4,42]. Despite these differences, the number of species associated with *A. palmata* was similar between both reefs (Limones = 64 species; Picudas = 51 species), as well as the composition of the associated communities. Taken together, these results suggest that the structure of *A. palmata* is capable of sustaining associated communities even in reefs with differing habitat quality, reinforcing its role as a foundational species. In agreement with [43], this evidence supports the notion that species diversity does not always respond directly to the overall condition of the reef and suggests that, at the colony scale, *A. palmata* may provide microhabitats that partially buffer the effects of reef degradation.

Interactions between corals and biological communities are influenced by numerous factors, including the specific ecological and behavioural requirements of each species [18,44]. Some organisms, such as the crustacean *D. acanthophora* and fishes of the family Gobiidae, are characterized by maintaining close associations with a single coral colony, within which they may remain for a large portion of their life cycle, as their ecological requirements are fulfilled within a reduced spatial scale at the colony level [45,46]. Consistent with this pattern, the present study observed that these groups used the structure of *A. palmata* as habitat. Complementarily, [47] documented that the crustacean *Carpilius corallinus*, a species with a similarly restricted distribution range, fed on the sessile polychaete *S. giganteus*. Although this specific interaction was not recorded in the present study, both species were observed associated with the structure of *A. palmata*, where *S. giganteus* stood out as one of the most abundant organisms. Additionally, *C. corallinus* was recorded feeding directly on the polyps of *A. palmata*, indicating direct use of this coral as a feeding site. This finding expands current knowledge of this species′ diet and highlights the diversity of potential trophic interactions associated with *A. palmata*.

By contrast, interactions associated with territorial behaviour generally involve species with varying degrees of substrate dependence and interest in remaining within a specific area of the reef structure [48]. A representative example is damselfishes (Pomacentridae), which are well known for their active defence of space [18]. Although these species may exhibit medium distribution ranges and relatively high mobility within the reef, their territorial behaviour is expressed through prolonged residence in specific areas where their ecological requirements are met [49]. By contrast, species of the family Haemulidae are characterized by wide nightly movements in search of food, while during the day they aggregate in small schools associated with coral structures, where they exhibit territorial behaviours primarily linked to resting periods [50,51]. This pattern was consistent with the findings of the present study, in which members of the family Pomacentridae exhibited territorial defence behaviours associated with *A. palmata* colonies. To a lesser extent, similar behaviours were recorded in some members of the families Haemulidae, Labridae and Labrisomidae, suggesting that the structure of *A. palmata* may facilitate spatial delimitation and the establishment of territories for species with different degrees of mobility and substrate dependence.

This spatial delimitation provided by the structure of *A. palmata* also allows different species to use it as a temporary refuge. In general terms, this behaviour has been associated with differences in mobility patterns and degrees of substrate dependence among taxonomic groups [52]. During periods of inactivity, mobile invertebrates tend to remain closely associated with the benthic substrate [52,53], whereas fishes, which generally exhibit broader distribution ranges, do not depend entirely on a single structure [50]. Despite these differences, both groups resort to coral structures during active periods, particularly when seeking resources or fulfilling other ecological requirements [53,54]. Accordingly, several invertebrate families (e.g. Carpiliidae, Palinuridae, Rhynchocinetidae, Cidaridae and Diadematidae) and different fish families (e.g. Labridae, Labrisomidae, Holocentridae and Pempheridae) use the highly complex structure of *A. palmata* as both refuge and feeding sites during active periods (supplementary material, appendix S1, table S3), suggesting complementary strategies to reduce predation risk through reduced spaces that limit the access of large epifauna [27].

Larger individuals were consistently found in the lower areas of the colony, whereas a greater number of individuals occupied the upper areas (figure 6). From a functional perspective, these findings are consistent with those of [44], who suggested that larger species require wide spaces that facilitate manoeuvrability needed to evade predators and with [55], who proposed that small reef species tend to avoid wide areas where they could be displaced by larger individuals. In crustaceans, families such as Palinuridae and Percnidae were primarily associated with the basal areas of the colony, whereas in fishes, families such as Haemulidae, Lutjanidae, Scaridae, Acanthuridae, Monacanthidae and Muraenidae, which are generally known to reach large body sizes, predominated in these zones. The present study suggests that these basal areas are sufficiently wide to allow manoeuvrability while simultaneously offering protection from larger predators.

By contrast, areas such as ‘Between branches’, although they may contain relatively wide spaces, offer reduced manoeuvrability owing to structural complexity, which limits the presence of large-bodied species [56]. This is consistent with experimental evidence from artificial *A. palmata* mimics, where the number of fish increased with structural complexity, yet cryptic taxa remained absent [57]. This is similar because complexity alone cannot substitute for the resources provided by living coral tissue, on which such species may critically depend. Accordingly, families Labridae, Holocentridae and Acanthuridae were more common in this area, whereas Labridae, Labrisomidae and Pempheridae predominated in the more exposed upper areas (‘On top’ and ‘Periphery’), typically as small- to medium-sized individuals. Labridae stood out for using both areas, reflecting its ability to exploit different microhabitats within the same colony.

Families such as Pomacentridae, Domeciidae, Rhynchocinetidae, Serpulidae and several echinoderms were distributed throughout the colony, indicating that the *A. palmata* structure provides available habitat space for species with different body sizes, mobility patterns and ecological strategies.

The decline of *A. palmata* in Caribbean reefs represents a critical concern, particularly in a region characterized by low coral diversity compared with the Indo-Pacific [14]. In the latter region, a single reef fish group can associate with up to 96 coral species, approximately 50 of which belong to the genus *Acropora* [14], suggesting high functional redundancy in terms of structure and habitat provision. By contrast, only two species of this genus have been reported in the Caribbean—*A. palmata* and *A. cervicornis* [58]—implying substantially lower functional redundancy. In this context, the loss of *A. palmata* represents not only the decline of a structurally dominant species but also the loss of ecological functions associated with its morphology, for which there are no clear functional equivalents in Caribbean reefs. The results of this study demonstrate a high diversity of associated fish and invertebrates, with more fish species than in other studies focusing on *A. cervicornis* [9]. The consistency with which *A. palmata* is used as a habitat, refuge and activity space by multiple taxonomic groups reinforces its role as a key functional species within these ecosystems. Consequently, the conservation of *A. palmata* is of particular relevance in the Caribbean compared with regions with higher coral diversity, not only owing to its structural contribution but also because of the ecological functions it supports at both colony and reef scales. The contribution of this study lies not in reaffirming the structural importance of *A. palmata*, but in resolving how that structure is used: by which taxa, through which behaviours and in which areas of the colony. Such patterns have rarely been documented simultaneously across taxonomically disparate groups or shown to persist across reef zones and reefs with different habitat quality.

## Supplementary Material



## Data Availability

Data supporting this study are openly available in the Figshare digital repository [59]. Supplementary material is available online [60].
