# Supplementary material for: Ecological role of Acropora palmata for its associated species in barrier reefs
Source: R Soc Open Sci. 2026 Oct 28;13(10):260900. doi: 10.1098/rsos.260900 (PMC13714379; doi:10.1098/rsos.260900)
Supplement: Supplementary file 1 [file rsos.260900.zip › rsos.260900/rsos260900_si_001.docx]

**Appendix S1**

**Supporting information for “Ecological role of *Acropora palmata* for its associated species in barrier reefs”**

Gandhi Germán Ramírez-Tapia, Anastazia Teresa Banaszak*****, Martha A. Gutiérrez-Aguirre, Lorenzo Álvarez-Filip

***Corresponding Author**

E-mail: banaszak@cmarl.unam.mx (ATB)

**Species accumulation curves**

**Figure S1.** Species accumulation curves for each reef (A) and each reef zone (B). Shading represents the 95% confidence intervals. The value of the non-parametric Chao1 estimator is also shown.

**List of species associated with *Acropora palmata***

**Table S1.** List of species associated with *Acropora palmata* in Limones and Picudas reefs, according to their density (individuals/m²) by reef zone. The average ± standard deviation for each species per reef zone is shown.

| **Species** | **Code** | **Limones** | | | **Total Limones** | **Picudas** | | | **Total Picudas** |
| --- | --- | --- | --- | --- | --- | --- | --- | --- | --- |
|  |  | **Fore** | **Crest** | **Back** |  | **Fore** | **Crest** | **Back** |  |
| *Abudefduf saxatilis* | ASAX |  | 0.13 ± 0.12 | 0.65 ± 1.28 | 0.34 ± 0.87 |  | 0.98 ± 1.59 | 0.17 ± 0.19 | 0.51 ± 1.11 |
| *Acanthurus chirurgus* | ACHI |  |  |  |  | 1.01 ± 1.43 | 0.13 ± 0.38 | 0.10 ± 0.17 | 0.22 ± 0.52 |
| *Acanthurus coeruleus* | ACOE | 0.15 ± 0.27 | 0.01 ± 0.03 | 1.02 ± 2.81 | 0.48 ± 1.87 | 5.47 ± 5.59 | 1.37 ± 1.98 | 1.88 ± 3.02 | 2.05 ± 2.97 |
| *Acanthurus tractus* | ATRA |  |  | 0.01 ± 0.01 | 0.002 ± 0.01 |  |  | 0.14 ± 0.40 | 0.06 ± 0.26 |
| *Anisotremus surinamensis* | ASUR |  |  |  |  |  |  | 0.02 ± 0.08 | 0.01 ± 0.05 |
| *Apogon maculatus* | AMAC |  |  | 0.21 ± 0.32 | 0.09 ± 0.23 |  | 0.33 ± 0.49 | 0.05 ± 0.15 | 0.17 ± 0.36 |
| *Astracme mucronata* | AMUC |  |  | 0.01 ± 0.02 | 0.003 ± 0.01 |  |  |  |  |
| *Bodianus rufus* | BRUF |  |  |  |  |  |  | 0.02 ± 0.06 | 0.01 ± 0.04 |
| *Cantherhines pullus* | CPUL |  | 0.01 ± 0.01 |  | 0.01 ± 0.01 |  |  |  |  |
| *Canthigaster rostrata* | CROS |  | 0.01 ± 0.02 | 0.02 ± 0.04 | 0.01 ± 0.03 |  |  |  |  |
| *Carpilius corallinus* | CCOR |  | 0.01 ± 0.01 |  | 0.002 ± 0.01 | 0.50 ± 0.71 |  |  | 0.05 ± 0.23 |
| *Cephalopholis cruentata* | CCRU |  |  | 0.02 ± 0.03 | 0.01 ± 0.02 |  |  |  |  |
| *Cerithium litteratum* | CLIT |  |  | 0.09 ± 0.26 | 0.04 ± 0.17 |  |  |  |  |
| *Chaetodon capistratus* | CCAP |  | 0.01 ± 0.01 | 0.10 ± 0.25 | 0.04 ± 0.17 | 0.25 ± 0.35 | 0.26 ± 0.76 | 0.16 ± 0.32 | 0.22 ± 0.54 |
| *Chaetodon striatus* | CSTR |  |  |  |  | 0.25 ± 0.35 |  |  | 0.02 ± 0.11 |
| **Species** | **Code** | **Limones** | | | **Total Limones** | **Picudas** | | | **Total Picudas** |
|  |  | **Fore** | **Crest** | **Back** |  | **Fore** | **Crest** | **Back** |  |
| *Chromis cyanea* | CCYA |  | 0.02 ± 0.04 | 0.06 ± 0.14 | 0.03 ± 0.10 |  | 0.13 ± 0.38 |  | 0.06 ± 0.25 |
| *Cinetorhynchus manningi* | CMAN |  |  | 0.02 ± 0.08 | 0.01 ± 0.05 | 0.25 ± 0.35 | 0.63 ± 0.80 | 0.41 ± 0.67 | 0.49 ± 0.69 |
| *Cinetorhynchus rigens* | CRIG |  |  | 0.01 ± 0.03 | 0.01 ± 0.02 |  |  |  |  |
| *Diadema antillarum* | DANT |  | 0.01 ± 0.014 | 0.39 ± 0.76 | 0.17 ± 0.53 |  | 0.32 ± 0.77 |  | 0.14 ± 0.52 |
| *Domecia acanthophora* | DACA | 3.09 ± 0.22 | 0.25 ± 0.20 | 1.05 ± 1.45 | 1.08 ± 1.37 | 2.33 ± 1.14 | 3.51 ± 4.06 | 2.33 ± 3.33 | 2.85 ± 3.43 |
| *Echinometra lucunter* | ELUC | 0.29 ± 0.25 | 0.01 ± 0.03 | 0.01 ± 0.03 | 0.06 ± 0.14 |  |  |  |  |
| *Echinometra viridis* | EVIR |  |  | 0.09 ± 0.25 | 0.04 ± 0.17 |  |  |  |  |
| *Elacatinus prochilos* | EPRO |  | 0.03 ± 0.05 | 0.02 ± 0.03 | 0.02 ± 0.03 |  |  | 0.29 ± 0.83 | 0.13 ± 0.55 |
| *Emblemariopsis sp.* | EMBL |  | 0.01 ± 0.01 | 0.01 ± 0.02 | 0.01 ± 0.01 |  |  |  |  |
| *Eucidaris tribuloides* | ETRI |  | 0.02 ± 0.03 | 0.30 ± 0.54 | 0.14 ± 0.38 | 0.50 ± 0.71 | 0.19 ± 0.38 |  | 0.14 ± 0.34 |
| *Gobioclinus kalisherae* | GKAL |  |  | 0.00 ± 0.02 | 0.003 ± 0.01 |  |  |  |  |
| *Haemulon aurolineatum* | HAUR |  | 0.004 ± 0.01 | 0.01 ± 0.02 | 0.01 ± 0.01 |  |  |  |  |
| *Haemulon carbonarium* | HCAR |  | 0.01 ± 0.01 | 0.22 ± 0.26 | 0.10 ± 0.20 |  | 0.05 ± 0.16 | 0.79 ± 1.67 | 0.38 ± 1.14 |
| *Haemulon chrysargireum* | HCHR |  | 0.01 ± 0.01 | 0.02 ± 0.05 | 0.01 ± 0.03 |  |  |  |  |
| *Haemulon flavolineatum* | HFLA |  | 0.03 ± 0.04 | 0.10 ± 0.13 | 0.05 ± 0.10 | 0.50 ± 0.71 | 0.10 ± 0.19 | 0.33 ± 0.53 | 0.25 ± 0.42 |
| *Haemulon macrostomum* | HMAC |  |  |  |  |  |  | 0.05 ± 0.09 | 0.02 ± 0.06 |
| *Haemulon plumierii* | HPLU |  |  | 0.01 ± 0.02 | 0.003 ± 0.01 |  |  |  |  |
| *Haemulon sciurus* | HSCI |  | 0.01 ± 0.03 | 0.01 ± 0.02 | 0.01 ± 0.02 |  |  |  |  |
| *Halichoeres bivittatus* | HBIV |  | 0.01 ± 0.01 | 0.21 ± 0.52 | 0.09 ± 0.35 | 0.75 ± 1.07 | 0.20 ± 0.37 | 0.42 ± 0.76 | 0.36 ± 0.63 |
| *Halichoeres garnoti* | HGAR |  | 0.01 ± 0.02 | 0.14 ± 0.26 | 0.06 ± 0.18 |  |  | 0.01 ± 0.03 | 0.01 ± 0.02 |
| *Halichoeres pictus* | HPIC |  |  | 0.03 ± 0.09 | 0.01 ± 0.06 |  |  |  |  |
| **Species** | **Code** | **Limones** | | | **Total Limones** | **Picudas** | | | **Total Picudas** |
|  |  | **Fore** | **Crest** | **Back** |  | **Fore** | **Crest** | **Back** |  |
| *Halichoeres radiatus* | HRAD |  | 0.01 ± 0.01 | 0.01 ± 0.02 | 0.01 ± 0.01 |  |  |  |  |
| *Hermodice carunculata* | HCARU |  |  | 0.11 ± 0.26 | 0.05 ± 0.17 |  |  |  |  |
| *Heteropriacanthus cruentatus* | HCRU |  |  | 0.09 ± 0.25 | 0.04 ± 0.17 |  |  |  |  |
| *Holacanthus ciliaris* | HCIL |  |  | 0.09 ± 0.26 | 0.04 ± 0.17 |  | 0.04 ± 0.13 | 0.05 ± 0.16 | 0.04 ± 0.13 |
| *Holocentrus adscensionis* | HADS |  |  |  |  |  | 0.13 ± 0.38 | 0.36 ± 0.78 | 0.22 ± 0.57 |
| *Lachnolaimus maximus* | LMAX |  |  |  |  |  |  | 0.14 ± 0.40 | 0.06 ± 0.26 |
| *Lutjanus apodus* | LAPO |  |  |  |  |  |  | 0.02 ± 0.08 | 0.01 ± 0.05 |
| *Lutjanus griseus* | LGRI |  |  | 0.01 ± 0.02 | 0.004 ± 0.01 |  |  |  |  |
| *Lutjanus jocu* | LJOC |  | 0.002 ± 0.01 | 0.02 ± 0.05 | 0.01 ± 0.03 |  |  | 0.05 ± 0.16 | 0.02 ± 0.11 |
| *Lutjanus mahogoni* | LMAO |  | 0.01 ± 0.01 |  | 0.004 ± 0.01 |  |  |  |  |
| *Malacoctenus aurolineatus* | MAUR | 0.21 ± 0.20 |  |  | 0.03 ± 0.10 |  | 0.18 ± 0.39 | 0.24 ± 0.51 | 0.19 ± 0.42 |
| *Malacoctenus triangulatus* | MTRI | 0.29 ± 0.25 | 0.01 ± 0.02 | 0.13 ± 0.26 | 0.11 ± 0.21 | 1.82 ± 1.86 |  |  | 0.20 ± 0.74 |
| *Microspathodon chrysurus* | MCHR | 0.60 ± 0.29 | 0.11 ± 0.10 | 0.08 ± 0.08 | 0.18 ± 0.23 | 0.50 ± 0.71 | 1.62 ± 3.26 | 0.99 ± 1.91 | 1.21 ± 2.46 |
| *Mithraculus sculptus* | MSCU |  |  |  |  |  | 0.04 ± 0.13 |  | 0.02 ± 0.08 |
| *Mithrax hispidus* | MHIS |  |  |  |  |  | 0.04 ± 0.13 |  | 0.02 ± 0.08 |
| *Mycteroperca venenosa* | MVEN |  |  | 0.01 ± 0.01 | 0.002 ± 0.01 |  |  |  |  |
| *Neoniphon vexillarium* | NVEX |  |  |  |  | 0.50 ± 0.72 |  |  | 0.05 ± 0.24 |
| *Nicholsina usta* | NUST |  | 0.01 ± 0.02 | 0.01 ± 0.04 | 0.01 ± 0.03 | 1.29 ± 0.40 |  |  | 0.14 ± 0.43 |
| *Octopus briareus* | OBRI |  |  | 0.01 ± 0.02 | 0.004 ± 0.01 |  | 0.14 ± 0.42 |  | 0.06 ± 0.28 |
| *Octopus vulgaris* | OVUL |  |  | 0.01 ± 0.02 | 0.003 ± 0.01 |  |  |  |  |
| *Ocyurus chrysurus* | OCHR |  |  | 0.09 ± 0.26 | 0.04 ± 0.17 |  |  |  |  |
| *Ophiuridae sp.* | OPHI |  |  |  |  | 0.25 ± 0.35 | 0.05 ± 0.16 |  | 0.05 ± 0.15 |
| **Species** | **Code** | **Limones** | | | **Total Limones** | **Picudas** | | | **Total Picudas** |
|  |  | **Fore** | **Crest** | **Back** |  | **Fore** | **Crest** | **Back** |  |
| *Palinurellus gundlachi* | PGUN |  |  |  |  |  |  | 0.07 ± 0.15 | 0.03 ± 0.10 |
| *Panulirus argus* | PARG |  |  | 0.02 ± 0.04 | 0.01 ± 0.02 |  |  |  |  |
| *Panulirus guttatus* | PGUT |  |  |  |  | 0.78 ± 1.11 | 0.34 ± 0.49 | 0.29 ± 0.26 | 0.36 ± 0.47 |
| *Pempheris schomburgkii* | PSCH |  | 0.09 ± 0.07 | 0.07 ± 0.13 | 0.071 ± 0.10 |  | 0.23 ± 0.66 | 0.13 ± 0.20 | 0.16 ± 0.45 |
| *Percnon gibbesi* | PGIB | 0.15 ± 0.26 | 0.01 ± 0.03 |  | 0.03 ± 0.10 | 1.57 ± 2.22 | 0.04 ± 0.13 | 0.04 ± 0.12 | 0.21 ± 0.74 |
| *Pomacanthus paru* | PPAR |  | 0.01 ± 0.01 | 0.01 ± 0.02 | 0.01 ± 0.02 |  |  | 0.14 ± 0.40 | 0.06 ± 0.26 |
| *Sargocentron bullisi* | SBUL | 0.29 ± 0.25 | 0.03 ± 0.02 | 0.14 ± 0.24 | 0.12 ± 0.20 | 2.35 ± 3.33 | 0.20 ± 0.38 |  | 0.35 ± 1.12 |
| *Sargocentron coruscum* | SCOR |  |  |  |  |  |  | 0.03 ± 0.08 | 0.01 ± 0.05 |
| *Sargocentron poco* | SPOC |  |  |  |  |  | 0.13 ± 0.38 | 0.11 ± 0.21 | 0.11 ± 0.28 |
| *Scarus iseri* | SISE |  | 0.01 ± 0.02 | 0.95 ± 2.36 | 0.42 ± 1.59 | 1.03 ± 0.75 | 0.72 ± 1.66 | 0.12 ± 0.18 | 0.49 ± 1.14 |
| *Scarus taeniopterus* | STAE |  |  | 0.01 ± 0.02 | 0.003 ± 0.01 | 0.50 ± 0.71 |  |  | 0.05 ± 0.23 |
| *Sparisoma aurofrenatum* | SAUR |  |  | 0.01 ± 0.01 | 0.002 ± 0.01 |  |  |  |  |
| *Sparisoma chrysopterum* | SCHR |  |  |  |  |  | 0.25 ± 0.41 |  | 0.11 ± 0.29 |
| *Sparisoma rubripinne* | SRUB |  | 0.01 ± 0.03 |  | 0.01 ± 0.02 |  |  |  |  |
| *Sparisoma viride* | SVIR |  | 0.04 ± 0.07 | 0.36 ± 0.73 | 0.17 ± 0.50 | 0.25 ± 0.35 | 0.14 ± 0.42 | 0.12 ± 0.21 | 0.15 ± 0.31 |
| *Spirobranchus giganteus* | SGIG | 6.10 ± 1.17 | 0.31 ± 0.47 | 0.79 ± 1.24 | 1.49 ± 2.33 | 7.30 ± 7.46 | 3.59 ± 4.40 | 2.00 ± 2.61 | 3.29 ± 4.09 |
| *Stegastes adustus* | SADU |  |  | 0.03 ± 0.10 | 0.01 ± 0.06 |  |  |  |  |
| *Stegastes leucostictus* | SLEU | 0.13 ± 0.23 |  |  | 0.02 ± 0.09 |  |  |  |  |
| *Stegastes partitus* | SPAR | 0.07 ± 0.13 | 0.02 ± 0.03 | 0.67 ± 1.34 | 0.32 ± 0.92 |  |  |  |  |
| *Stegastes planifrons* | SPLA | 0.29 ± 0.25 | 0.15 ± 0.10 | 1.44 ± 1.96 | 0.75 ± 1.41 | 2.07 ± 1.50 | 2.08 ± 2.53 | 0.74 ± 0.64 | 1.48 ± 1.85 |
| **Species** | **Code** | **Limones** | | | **Total Limones** | **Picudas** | | | **Total Picudas** |
|  |  | **Fore** | **Crest** | **Back** |  | **Fore** | **Crest** | **Back** |  |
| *Thalassoma bifasciatum* | TBIF | 6.15 ± 6.97 | 0.08 ± 0.08 | 0.85 ± 1.79 | 1.44 ± 3.44 | 7.24 ± 3.08 | 3.23 ± 3.70 | 3.56 ± 4.92 | 3.82 ± 4.21 |
| *Tigrigobius dilepis* | TDIL |  |  | 0.01 ± 0.02 | 0.004 ± 0.01 |  |  | 0.20 ± 0.41 | 0.08 ± 0.28 |
| *Tripneustes ventricosus* | TVEN |  |  | 0.01 ± 0.02 | 0.003 ± 0.01 |  |  |  |  |

**Most common habitat usage behaviors within coral colonies**

**Table S2**. Description of the most common habitat usage behaviors within coral colonies, based on a review of the literature, and applied across major taxonomic groups (fish, crustacean, echinoderm, and polychaeta).

| **Group** | **Family**  **(common name)** | **Activity period** | **Feeding behavior** | **Habitat-use category** | **Ecological traits relevant to habitat-use classification** | **Reference** |
| --- | --- | --- | --- | --- | --- | --- |
| Fish | Pomacentridae (Damselfish) | Diurnal | Omnivorous; algal farming, plankton, small invertebrates | Habitat, Refuge, Territorial-Behavior | Damselfishes establish and actively defend territories within branching corals, using the coral structure for nesting, breeding, and refuge | [1–4] |
| Fish | Gobiidae (Goby) | Diurnal–Nocturnal | Small benthic invertebrates | Habitat | Many gobies show strong fidelity to individual coral colonies and remain closely associated with live coral tissue throughout multiple life stages | [5–7] |
| Fish | Scaridae (Parrotfish) | Diurnal | Herbivorous scraper | Feeding Site | Parrotfish do not reside within coral colonies but frequently visit reef substrates, including coral surfaces, during grazing and scraping activities | [8–10] |
| Fish | Labridae (Wrasse) | Diurnal–Nocturnal | Small benthic invertebrates | Refuge | Wrasses commonly use coral structures as temporary refuge during resting periods or when avoiding predators | [11–13] |
| **Group** | **Family**  **(common name)** | **Activity period** | **Feeding behavior** | **Habitat-use category** | **Ecological traits relevant to habitat-use classification** | **Reference** |
| Fish | Haemulidae (Grunt) | Diurnal–Nocturnal | Benthic invertebrates | Refuge | Grunts during the day regularly aggregate in structurally complex coral formations and disperse at night in search of food requirements. | [14–17] |
| Fish | Lutjanidae (Snapper) | Diurnal–Nocturnal | Benthic invertebrates | Refuge | Snappers aggregate near structurally complex coral formations during daylight hours and migrate nocturnally to feed in adjacent habitats | [14–16,18] |
| Crustacean | Domeciidae | Nocturnal | Omnivorous | Habitat | Domeciid crabs live permanently on coral colonies and exhibit high host specificity, particularly toward branching corals such as *Acropora* spp. | [19–21] |
| Crustacean | Mithracidae | Nocturnal | Omnivorous; algae and detritus | Refuge, Feeding Site | Mithracid crabs use coral structural complexity as refuge and forage opportunistically on organisms associated with coral colonies | [22–25] |
| Crustacean | Rhynchocinetidae | Nocturnal | Omnivorous | Refuge | These cryptic shrimps use coral and reef structural complexity as temporary shelter during inactive periods | [24,26–28] |
| Crustacean | Carpiliidae | Nocturnal | Carnivorous; mollusks and echinoderms | Refuge, Feeding Site | Carpiliid crabs rely on coral and reef structures for protection and as feeding grounds but do not reside permanently within coral colonies | [24,29,30] |
| Polychaete | Serpulidae (Serpulid worm) | Diurnal–Nocturnal | Suspension feeder | Habitat | Serpulid worms permanently inhabit calcareous tubes embedded within coral skeletons, representing a direct and long-term association with the coral structure | [31–35] |
| Polychaete | Amphinomidae (Fireworm) | Nocturnal | Carnivorous; coral tissue and associated organisms | Feeding Site | Fireworms feed on coral tissue or associated organisms but occur only temporarily on coral colonies and are not permanent residents | [36–38] |
| **Group** | **Family**  **(common name)** | **Activity period** | **Feeding behavior** | **Habitat-use category** | **Ecological traits relevant to habitat-use classification** | **Reference** |
| Echinoderm | Diadematidae (Long-spined sea urchin) | Nocturnal | Omnivorous grazer | Refuge, Feeding Site | Long-spined sea urchins use coral structures and reef crevices as refuge during inactive periods and forage in surrounding areas | [36,39–43] |
| Echinoderm | Ophiuridae | Nocturnal | Small invertebrates | Refuge | Brittle stars use coral structural complexity as shelter, remaining hidden inside holes or crevices of coral during daylight hours | [36,44–46] |
| Echinoderm | Cidaridae (Pencil sea urchin) | Nocturnal | Small benthic invertebrates | Refuge, Feeding Site | Pencil sea urchins intermittently occupy reef and coral structures while foraging and using coral complexity as temporary refuge | [47–50] |

**Dominant habitat use behaviors and dominant colony areas by taxonomic family**

**Table S3.** Dominant behavior and dominant colony area associated with *Acropora palmata* for each taxonomic family, summarized independently based on relative frequencies across all observations. For each descriptor, the number of observations supporting the dominant category is reported relative to the total number of observations per family.

| **Group** | **Family** | **Dominant habitat use behavior** | $\mathbf{n/Total}$ | **Dominant colony area** | $\mathbf{n/Total}$ |
| --- | --- | --- | --- | --- | --- |
| Fish | Acanthuridae | Refuge | $59/79$ | Base | $28/79$ |
| Fish | Apogonidae | Refuge | $6/10$ | Periphery | $4/10$ |
| Fish | Chaenopsidae | Refuge | $1/1$ | On top | $1/1$ |
| Fish | Chaetodontidae | Feeding Site | $8/12$ | Inside | $4/12$ |
| Fish | Gobiidae | Habitat | $15/19$ | On top | $11/19$ |
| Fish | Haemulidae | Refuge | $95/96$ | Base | $48/96$ |
| Fish | Holocentridae | Refuge | $34/41$ | Base | $13/41$ |
| Fish | Labridae | Refuge | $245/255$ | Periphery | $125/255$ |
| Fish | Labrisomidae | Refuge | $14/28$ | On top | $15/28$ |
| Fish | Lutjanidae | Refuge | $13/13$ | Inside | $7/13$ |
| Fish | Monacanthidae | Refuge | $3/3$ | Base | $3/3$ |
| Fish | Muraenidae | Refuge | $2/2$ | Base | $2/2$ |
| Fish | Pempheridae | Refuge | $33/44$ | Periphery | $28/44$ |
| Fish | Pomacanthidae | Refuge | $1/1$ | Periphery | $1/1$ |
| Fish | Pomacentridae | Territorial Behavior | $197/334$ | Base | $130/334$ |
| Fish | Priacanthidae | Refuge | $1/1$ | Base | $1/1$ |
| Fish | Scaridae | Refuge | $89/94$ | Base | $30/94$ |
| Fish | Serranidae | Refuge | $4/4$ | Base | $2/4$ |
| Fish | Tetraodontidae | Refuge | $7/9$ | Base | $8/9$ |
| Crustacean | Carpiliidae | Feeding Site | $3/5$ | Base | $2/5$ |
| Crustacean | Domeciidae | Habitat | $224/254$ | On top | $152/254$ |
| Crustacean | Mithracidae | Refuge | $1/2$ | Base | $1/2$ |
| Crustacean | Palinuridae | Refuge | $18/19$ | Base | $19/19$ |
| Crustacean | Percnidae | Feeding Site | $6/8$ | Base | $7/8$ |
| Crustacean | Rhynchocinetidae | Refuge | $15/26$ | On top | $9/26$ |
| Polychaete | Amphinomidae | Feeding Site | $4/4$ | On top | $4/4$ |
| Polychaete | Serpulidae | Habitat | $247/247$ | On top | $187/247$ |
| Echinoderm | Cidaridae | Refuge | $12/15$ | Base | $12/15$ |
| Echinoderm | Diadematidae | Feeding Site | $9/13$ | Base | $8/13$ |
| Echinoderm | Echinometridae | Feeding Site | $5/7$ | Base | $6/7$ |
| Echinoderm | Gorgonocephalidae | Refuge | $1/1$ | On top | $1/1$ |
| **Group** | **Family** | **Dominant habitat use behavior** | $\mathbf{n/Total}$ | **Dominant colony area** | $\mathbf{n/Total}$ |
| Echinoderm | Ophiuridae | Refuge | $2/2$ | Base | $2/2$ |
| Echinoderm | Toxopneustidae | Refuge | $1/1$ | Base | $1/1$ |
| Mollusk | Ceritiidae | Feeding Site | $1/1$ | On top | $1/1$ |
| Mollusk | Octoporidae | Refuge | $3/3$ | Base | $3/3$ |

**Table S4.** Summary of the number of *A. palmata* colonies and the number of associated individuals per transect.

| **Transect ID** | **Number of colonies** | **Number of associated individuals** |
| --- | --- | --- |
| Limones Fore 1 | 4 | 57 |
| Limones Fore 2 | 5 | 49 |
| Limones Fore 3 | 2 | 38 |
| Limones Crest 1 | 2 | 39 |
| ^a^ Limones Crest 2 | 1 | 34 |
| Limones Crest 3 | 4 | 56 |
| Limones Crest 4 | 3 | 47 |
| ^a^ Limones Crest 5 | 1 | 62 |
| ^a^ Limones Crest 6 | 1 | 51 |
| Limones Crest 7 | 3 | 58 |
| Limones Back 1 | 5 | 58 |
| Limones Back 2 | 8 | 31 |
| Limones Back 3 | 5 | 74 |
| Limones Back 4 | 3 | 76 |
| Limones Back 5 | 3 | 36 |
| Limones Back 6 | 3 | 95 |
| Limones Back 7 | 3 | 76 |
| Limones Back 8 | 3 | 65 |
| Picudas Fore 1 | 16 | 50 |
| Picudas Fore 2 | 15 | 34 |
| Picudas Crest 1 | 6 | 8 |
| Picudas Crest 2 | 4 | 31 |
| Picudas Crest 3 | 8 | 36 |
| Picudas Crest 4 | 16 | 45 |
| Picudas Crest 5 | 8 | 39 |
| Picudas Crest 6 | 10 | 39 |
| Picudas Crest 7 | 6 | 29 |
| Picudas Crest 8 | 5 | 44 |
| Picudas Back 1 | 3 | 30 |
| Picudas Back 2 | 3 | 42 |
| Picudas Back 3 | 5 | 44 |
| Picudas Back 4 | 6 | 19 |
| Picudas Back 5 | 4 | 49 |
| Picudas Back 6 | 6 | 37 |
| Picudas Back 7 | 10 | 32 |
| Picudas Back 8 | 7 | 41 |

^a^ At Limones crest, *A. palmta* formed dense, continuous patches, making it impossible to distinguish the boundaries of each individual colony. In these cases, as a precaution, each patch was recorded as a single colony.

**GLMM 1: Habitat use behaviors**

**Table S5.** Estimated Parameters of the Fixed Effects from the Generalized Linear Mixed Model assessing the effect of behavior and taxonomic group, as well as their interactions, on the habitat use of species associated with *Acropora palmata*. Fixed effects are presented as standard deviations. Number of observations: 578; Groups: 4; Reef zones: 3; Reefs: 2.

| **Predictor** | **Estimate** | **Std. Error** | **t value** | **Pr(>\|z\|)** |
| --- | --- | --- | --- | --- |
| **Fixed effects:** |  |  |  |  |
| (Intercept) | 0.494 | 0.160 | 3.085 | 0.002 |
| **Behavior** |  |  |  |  |
| Territorial behavior | 0.260 | 0.152 | 1.716 | 0.086 |
| Habitat | 1.053 | 0.200 | 5.270 | 0.000 |
| Refuge | -0.167 | 0.212 | -0.791 | 0.429 |
| **Group** |  |  |  |  |
| Echinoderm | -0.236 | 0.260 | -0.910 | 0.363 |
| Fish | -0.098 | 0.189 | -0.520 | 0.603 |
| Polychaete | -0.531 | 0.390 | -1.362 | 0.173 |
| **Interaction Behavior: Group** |  |  |  |  |
| Refuge: Echinoderm | -0.070 | 0.333 | -0.209 | 0.834 |
| Habitat: Fish | -1.052 | 0.288 | -3.648 | 0.000 |
| Refuge: Fish | 0.348 | 0.241 | 1.444 | 0.149 |
| Habitat: Polychaete | 0.704 | 0.428 | 1.644 | 0.100 |
| **Random effects:** | **Name** | **Variance** | **Std. Dev.** |  |
| Reef zone | (Intercept) | 0 | 0 |  |
| Reef | (Intercept) | 0.001 | 0.042 |  |
| Residual |  | 0.507 | 0.712 |  |

**Table S6.** Conditional and Marginal *R^2^* values with the differences attributed to random effects from the Generalized Linear Mixed Model on the habitat use of species associated with *Acropora palmata*.

| **Description** | **Value** |
| --- | --- |
| Marginal *R^2^* (Fixed effects only) | 0.222 |
| Conditional *R^2^* (Fixed and random effects) | 0.225 |
| Difference attributed to Random effects | 0.002 |

**GLMM 2: Colony area effects on the number of individuals**

**Table S7.** Estimated parameters of the fixed effects from the Generalized Linear Mixed Model assessing the effect of colony area locations and taxonomic group interactions on the number of individuals associated with *Acropora palmata*. Fixed effects are presented as standard deviations. Number of observations: 703; Groups: 4; Reef zones: 3; Reefs: 2.

| **Predictor** | **Estimate** | **Std. Error** | **t value** | **Pr(>\|z\|)** |
| --- | --- | --- | --- | --- |
| **Fixed effects:** |  |  |  |  |
| (Intercept) | 0.355 | 0.141 | 2.514 | 0.012 |
| **Area of the Colony** |  |  |  |  |
| Inside | -0.353 | 0.663 | -0.532 | 0.595 |
| Between branches | 0.405 | 0.172 | 2.358 | 0.018 |
| Periphery | 0.642 | 0.315 | 2.039 | 0.042 |
| On top | 0.711 | 0.162 | 4.396 | 0.000 |
| **Group** |  |  |  |  |
| Echinoderm | -0.211 | 0.180 | -1.175 | 0.240 |
| Fish | -0.002 | 0.129 | -0.016 | 0.988 |
| Polychaete | 0.364 | 0.396 | 0.920 | 0.358 |
| **Interaction Area of the Colony: Group** |  |  |  |  |
| Inside: Echinoderm | 0.211 | 0.937 | 0.225 | 0.822 |
| Between branches: Echinoderm | -0.546 | 0.686 | -0.796 | 0.426 |
| Periphery: Echinoderm | -0.847 | 0.574 | -1.474 | 0.140 |
| On top: Echinoderm | -0.777 | 0.359 | -2.161 | 0.031 |
| Inside: Fish | 0.397 | 0.668 | 0.593 | 0.553 |
| Between branches: Fish | -0.369 | 0.196 | -1.876 | 0.061 |
| Periphery: Fish | -0.199 | 0.329 | -0.604 | 0.546 |
| On top: Fish | -0.563 | 0.179 | -3.137 | 0.002 |
| Inside: Polychaete | -0.122 | 0.808 | -0.150 | 0.881 |
| Between branches: Polychaete | -0.584 | 0.462 | -1.264 | 0.206 |
| Periphery: Polychaete | -0.219 | 0.558 | -0.392 | 0.695 |
| On top: Polychaete | -0.113 | 0.426 | -0.265 | 0.791 |
| **Random effects:** | **Name** | **Variance** | **Std. Dev.** |  |
| Reef zone | (Intercept) | 0.003 | 0.054 |  |
| Reef | (Intercept) | 0.003 | 0.057 |  |
| Residual |  | 0.425 | 0.652 |  |

**Table S8.** Conditional and Marginal *R^2^* values with the difference attributed to random effects from the Generalized Linear Mixed Model on the number of individuals associated with *Acropora palmata*.

| **Description** | **Value** |
| --- | --- |
| Marginal *R^2^* (Fixed effects only) | 0.158 |
| Conditional *R^2^* (Fixed and random effects) | 0.170 |
| Difference attributed to random effects | 0.011 |

**GLMM 3: Colony area effects on the size of individuals**

**Table S9.** Estimated parameters of the fixed effects from the Generalized Linear Mixed Model assessing the effect of colony area locations and taxonomic group interactions on the size of individuals associated with *Acropora palmata*. Fixed effects are presented as standard deviations. Number of observations: 1650 ^a^; Groups: 4; Reef zones: 3; Reefs: 2.

| **Predictor** | **Estimate** | **Std. Error** | **t value** | **Pr(>\|z\|)** |
| --- | --- | --- | --- | --- |
| **Fixed effects:** |  |  |  |  |
| (Intercept) | 1.929 | 0.133 | 14.547 | < 2e-16 |
| **Area of the Colony** |  |  |  |  |
| Inside | 0.277 | 0.568 | 0.488 | 0.626 |
| Between branches | -1.349 | 0.101 | -13.297 | < 2e-16 |
| Periphery | -1.554 | 0.162 | -9.562 | < 2e-16 |
| On top | -1.474 | 0.092 | -16.062 | < 2e-16 |
| **Group** |  |  |  |  |
| Echinoderm | 0.113 | 0.132 | 0.858 | 0.391 |
| Fish | 0.289 | 0.086 | 3.356 | 0.001 |
| Polychaete | -1.725 | 0.244 | -7.065 | 0.000 |
| **Interaction Area of the Colony: Group** |  |  |  |  |
| Inside: Echinoderm | -0.218 | 0.806 | -0.271 | 0.786 |
| Between branches: Echinoderm | 1.290 | 0.581 | 2.222 | 0.026 |
| Periphery: Echinoderm | 1.455 | 0.442 | 3.290 | 0.001 |
| On top: Echinoderm | 1.858 | 0.269 | 6.908 | 0.000 |
| Inside: Fish | -0.233 | 0.571 | -0.408 | 0.683 |
| Between branches: Fish | 1.223 | 0.120 | 10.228 | < 2e-16 |
| Periphery: Fish | 1.200 | 0.171 | 7.031 | 0.000 |
| On top: Fish | 1.003 | 0.103 | 9.718 | < 2e-16 |
| Inside: Polychaete | -0.116 | 0.645 | -0.180 | 0.857 |
| Between branches: Polychaete | 1.289 | 0.281 | 4.583 | 0.000 |
| Periphery: Polychaete | 1.499 | 0.302 | 4.963 | 0.000 |
| On top: Polychaete | 1.474 | 0.253 | 5.837 | 0.000 |
| **Random effects:** | **Name** | **Variance** | **Std. Dev.** |  |
| Reef zone | (Intercept) | 0.004 | 0.070 |  |
| Reef | (Intercept) | 0.004 | 0.066 |  |
| Residual |  | 0.317 | 0.563 |  |

^a^ The total number of associated individuals recorded across all transects was 1651 (Table S4). One record was excluded during model fitting owing to an incomplete entry, resulting in the 1650 observations analysed here.**Table S10.** Conditional and Marginal *R^2^* values with the difference attributed to random effects from the Generalized Linear Mixed Model on the size of individuals associated with *Acropora palmata*.

| **Description** | **Value** |
| --- | --- |
| Marginal *R^2^* (Fixed effects only) | 0.661 |
| Conditional *R^2^* (Fixed and random effects) | 0.671 |
| Difference attributed to random effects | 0.010 |

**References**

1.	Chase T, Pratchett M, Hoogenboom M. 2020 Behavioral trade-offs and habitat associations of coraldwelling damselfishes (family Pomacentridae). *Mar. Ecol. Prog. Ser.* **633**, 141–156. (doi:10.3354/meps13167)

2.	Fishelson L. 1970 Behaviour and ecology of a population of Abudefduf saxatilis (Pomacentridae, Teleostei) at Eilat (Red Sea). *Anim. Behav.* **18**, 225–237. (doi:10.1016/S0003-3472(70)80032-X)

3.	Jones KMM. 2005 The effect of territorial damselfish (family Pomacentridae) on the space use and behaviour of the coral reef fish, Halichoeres bivittatus (Bloch, 1791) (family Labridae). *J. Exp. Mar. Biol. Ecol.* **324**, 99–111. (doi:10.1016/j.jembe.2005.04.009)

4.	Pratchett MS, Coker DJ, Jones GP, Munday PL. 2012 Specialization in habitat use by coral reef damselfishes and their susceptibility to habitat loss. *Ecol. Evol.* **2**, 2168–2180. (doi:10.1002/ece3.321)

5.	Brandl SJ *et al.* 2019 Demographic dynamics of the smallest marine vertebrates fuel coral reef ecosystem functioning. *Science* **364**, 1189–1192. (doi:10.1126/science.aav3384)

6.	Munday PL. 2004 Habitat loss, resource specialization, and extinction on coral reefs. *Glob. Change Biol.* **10**, 1642–1647. (doi:10.1111/j.1365-2486.2004.00839.x)

7.	Pratchett MS, Thompson CA, Hoey AS, Cowman PF, Wilson SK. 2018 Effects of coral bleaching and coral loss on the structure and function of reef fish assemblages. In *Coral Bleaching* (eds MJH Van Oppen, JM Lough), pp. 265–293. Cham: Springer International Publishing. (doi:10.1007/978-3-319-75393-5_11)

8.	Bellwood DR, Hughes TP, Folke C, Nyström M. 2004 Confronting the coral reef crisis. *Nature* **429**, 827–833. (doi:10.1038/nature02691)

9.	Francini-Filho RB, Moura RL, Ferreira CM, Coni EOC. 2008 Live coral predation by parrotfishes (Perciformes: Scaridae) in the Abrolhos Bank, eastern Brazil, with comments on the classification of species into functional groups. *Neotropical Ichthyol.* **6**, 191–200. (doi:10.1590/S1679-62252008000200006)

10.	Mumby PJ. 2006 The impact of exploiting grazers (Scaridae) on the dynamics of Caribbean coral reefs. *Ecol. Appl.* **16**, 747–769. (doi:10.1890/1051-0761(2006)016[0747:TIOEGS]2.0.CO;2)

11.	Fulton C, Bellwood D, Wainwright P. 2001 The relationship between swimming ability and habitat use in wrasses (Labridae). *Mar. Biol.* **139**, 25–33. (doi:10.1007/s002270100565)

12.	Fulton C, Bellwood D. 2002 Patterns of foraging in labrid fishes. *Mar. Ecol. Prog. Ser.* **226**, 135–142. (doi:10.3354/meps226135)

13.	Westneat MW, Alfaro ME, Wainwright PC, Bellwood DR, Grubich JR, Fessler JL, Clements KD, Smith LL. 2005 Local phylogenetic divergence and global evolutionary convergence of skull function in reef fishes of the family Labridae. *Proc. R. Soc. B Biol. Sci.* **272**, 993–1000. (doi:10.1098/rspb.2004.3013)

14.	Clark RD, Pittman S, Caldow C, Christensen J, Roque B, Appeldoorn RS, Monaco ME. 2009 Nocturnal fish movement and trophic flow across habitat boundaries in a coral reef ecosystem (SW Puerto Rico). *Caribb. J. Sci.* **45**, 282–303. (doi:10.18475/cjos.v45i2.a15)

15.	Cocheret de la Morinière E, Pollux BJA, Nagelkerken I, van der Velde G. 2003 Diet shifts of Caribbean grunts (Haemulidae) and snappers (Lutjanidae) and the relation with nursery-to-coral reef migrations. *Estuar. Coast. Shelf Sci.* **57**, 1079–1089. (doi:10.1016/S0272-7714(03)00011-8)

16.	Nagelkerken I, Dorenbosch M, Verberk W, Cocheret De La Morinière E, Van Der Velde G. 2000 Day-night shifts of fishes between shallow-water biotopes of a Caribbean bay, with emphasis on the nocturnal feeding of Haemulidae and Lutjanidae. *Mar. Ecol. Prog. Ser.* **194**, 55–64. (doi:10.3354/meps194055)

17.	Verweij MC, Nagelkerken I. 2007 Short and long-term movement and site fidelity of juvenile Haemulidae in back-reef habitats of a Caribbean embayment. *Hydrobiologia* **592**, 257–270. (doi:10.1007/s10750-007-0772-9)

18.	Luo J, Serafy J, Sponaugle S, Teare P, Kieckbusch D. 2009 Movement of gray snapper *Lutjanus griseus* among subtropical seagrass, mangrove, and coral reef habitats. *Mar. Ecol. Prog. Ser.* **380**, 255–269. (doi:10.3354/meps07911)

19.	Glynn PW. 2025 Symbiosis: microorganisms to the megafauna. In *Coral Reef Resilience in the Anthropocene*, pp. 295–320. Cham: Springer Nature Switzerland. (doi:10.1007/978-3-031-81421-1_11)

20.	Hoeksema BW, Fransen CHJM. 2011 Space partitioning by symbiotic shrimp species cohabitating in the mushroom coral Heliofungia actiniformis at Semporna, eastern Sabah. *Coral Reefs* **30**, 519–519. (doi:10.1007/s00338-011-0736-4)

21.	van der Meij SET, Bravo H, Scholten YJH, Dromard CR. 2022 Host use of the elkhorn coral crab *Domecia acanthophora* (Brachyura : Domeciidae), with a phylogeny of the genus. (doi:10.21411/CBM.A.946E2CEE)

22.	Butler MJ, Mojica AM. 2012 Herbivory by the Caribbean king crab on coral patch reefs. *Mar. Biol.* **159**, 2697–2706. (doi:10.1007/s00227-012-2027-1)

23.	Dubé MK, Barradas-Ortiz C, Negrete-Soto F, Álvarez-Filip L, Lozano-Álvarez E, Briones-Fourzán P. 2023 A shift in communities of conspicuous macrocrustaceans associated with caribbean coral reefs following a series of environmental stressors. *Diversity* **15**, 809. (doi:10.3390/d15070809)

24.	Giraldes BW, Coelho Filho PA, Smyth DM, Coelho PA. 2017 The nocturnal zonation of decapods in the subtidal zone within the reef seascape—abiotic factors defining habitats. *ICES J. Mar. Sci.* **74**, 2180–2190. (doi:10.1093/icesjms/fsx055)

25.	González-Gómez R, Briones-Fourzán P, Álvarez-Filip L, Lozano-Álvarez E. 2018 Diversity and abundance of conspicuous macrocrustaceans on coral reefs differing in level of degradation. *PeerJ* **6**, e4922. (doi:10.7717/peerj.4922)

26.	Bauer R, Okuno J, Thiel M. 2014 Inferences on mating and sexual systems of two Pacific Cinetorhynchus shrimps (Decapoda, Rhynchocinetidae) based on sexual dimorphism in body size and cheliped weaponry. *ZooKeys* **457**, 187–209. (doi:10.3897/zookeys.457.6512)

27.	Ory NC, Dudgeon D, Dumont CP, Miranda L, Thiel M. 2012 Effects of predation and habitat structure on the abundance and population structure of the rock shrimp Rhynchocinetes typus (Caridea) on temperate rocky reefs. *Mar. Biol.* **159**, 2075–2089. (doi:10.1007/s00227-012-1994-6)

28.	Ory NC, Dudgeon D, Duprey N, Thiel M. 2014 Effects of predation on diel activity and habitat use of the coral-reef shrimp *Cinetorhynchus hendersoni* (Rhynchocinetidae). *Coral Reefs* **33**, 639–650. (doi:10.1007/s00338-014-1163-0)

29.	Muller E, De Gier W, Ten Hove HA, Van Moorsel GWNM, Hoeksema BW. 2020 Nocturnal predation of christmas tree worms by a batwing coral crab at Bonaire (Southern Caribbean). *Diversity* **12**, 455. (doi:10.3390/d12120455)

30.	Wetzer R, Martin JW, Trautwein SE. 2003 Phylogenetic relationships within the coral crab genus *Carpilius* (Brachyura, Xanthoidea, Carpiliidae) and of the Carpiliidae to other xanthoid crab families based on molecular sequence data. *Mol. Phylogenet. Evol.* **27**, 410–421. (doi:10.1016/S1055-7903(03)00021-6)

31.	Hoeksema BW, Wels D, Van Der Schoot RJ, Ten Hove HA. 2019 Coral injuries caused by *Spirobranchus opercula* with and without epibiotic turf algae at Curaçao. *Mar. Biol.* **166**, 60. (doi:10.1007/s00227-019-3504-6)

32.	Hoeksema BW, Ten Hove HA. 2014 First record of a christmas tree worm in a mushroom coral (Loyalty Islands, Southwest Pacific). *Coral Reefs* **33**, 717–717. (doi:10.1007/s00338-014-1175-9)

33.	Hutchings P. 2011 Bioerosion. In *Encyclopedia of Modern Coral Reefs* (ed D Hopley), pp. 139–156. Dordrecht: Springer Netherlands. (doi:10.1007/978-90-481-2639-2_5)

34.	Marsden JR. 1987 Coral preference behaviour by planktotrophic larvae of *Spirobranchus giganteus corniculatus* (Serpulidae: Polychaeta). *Coral Reefs* **6**, 71–74. (doi:10.1007/BF00301376)

35.	Perry O, Sapir Y, Perry G, Ten Hove H, Fine M. 2018 Substrate selection of Christmas tree worms ( *Spirobranchus* spp.) in the Gulf of Eilat, Red Sea. *J. Mar. Biol. Assoc. U. K.* **98**, 791–799. (doi:10.1017/S0025315416002022)

36.	Glynn PW, Enochs IC. 2011 Invertebrates and their roles in coral reef ecosystems. In *Coral Reefs: An Ecosystem in Transition* (eds Z Dubinsky, N Stambler), pp. 273–325. Dordrecht: Springer Netherlands. (doi:10.1007/978-94-007-0114-4_18)

37.	Schulze A, Grimes CJ, Rudek TE. 2017 Tough, armed and omnivorous: *Hermodice carunculata* (Annelida: Amphinomidae) is prepared for ecological challenges. *J. Mar. Biol. Assoc. U. K.* **97**, 1075–1080. (doi:10.1017/S0025315417000091)

38.	Wolf AT, Nugues MM, Wild C. 2014 Distribution, food preference, and trophic position of the corallivorous fireworm *Hermodice carunculata* in a Caribbean coral reef. *Coral Reefs* **33**, 1153–1163. (doi:10.1007/s00338-014-1184-8)

39.	Bodmer MDV, Wheeler PM, Anand P, Cameron SE, Hintikka S, Cai W, Borcsok AO, Exton DA. 2021 The ecological importance of habitat complexity to the Caribbean coral reef herbivore *Diadema antillarum*: three lines of evidence. *Sci. Rep.* **11**, 9382. (doi:10.1038/s41598-021-87232-9)

40.	de Breuyn M, van der Last AJ, Klokman OJ, Hylkema A. 2023 Diurnal predators of restocked lab-reared and wild *Diadema antillarum* near artificial reefs in Saba. *PeerJ* **11**, e16189. (doi:10.7717/peerj.16189)

41.	Dunn R, Altieri A, Miller K, Yeager M, Hovel K. 2018 Contrasting behavioral responses to predatory risk cues reflect different foraging strategies in two Caribbean sea urchins. *Mar. Ecol. Prog. Ser.* **604**, 187–198. (doi:10.3354/meps12733)

42.	Kintzing MD, Butler MJ. 2014 The influence of shelter, conspecifics, and threat of predation on the behavior of the long-spined sea urchin ( *Diadema antillarum* ). *J. Shellfish Res.* **33**, 781–785. (doi:10.2983/035.033.0312)

43.	Tuya F, Martin JA, Luque A. 2004 Patterns of nocturnal movement of the long-spined sea urchin *Diadema antillarum* (Philippi) in Gran Canaria (the Canary Islands, central East Atlantic Ocean). *Helgol. Mar. Res.* **58**, 26–31. (doi:10.1007/s10152-003-0164-0)

44.	Drolet D, Himmelman JH, Rochette R. 2004 Use of refuges by the ophiuroid *Ophiopholis aculeata*: contrasting effects of substratum complexity on predation risk from two predators. *Mar. Ecol. Prog. Ser.* **284**, 173/183.

45.	Drolet D, Himmelman JH, Rochette R. 2004 Effect of light and substratum complexity on microhabitat selection and activity of the ophiuroid *Ophiopholis aculeata*. *J. Exp. Mar. Biol. Ecol.* **313**, 139–154. (doi:10.1016/j.jembe.2004.08.004)

46.	Turner RL, Boucher JM. 2010 Loss of arm spines in the basketstar *Astrophyton muricatum* (Echinodermata: Ophiuroidea: Gorgonocephalidae), with a key to West Indian basketstars. *Proc. Biol. Soc. Wash.* **123**, 17–26. (doi:10.2988/09-10.1)

47.	Dee LE, Witman JD, Brandt M. 2012 Refugia and top-down control of the pencil urchin Eucidaris galapagensis in the Galápagos Marine Reserve. *J. Exp. Mar. Biol. Ecol.* **416–417**, 135–143. (doi:10.1016/j.jembe.2012.02.016)

48.	Lessios HA, Kessing BD, Robertson DR, Paulay G. 1999 Phylogeography of the pantropical sea urchin *Eucidaris* in relation to land barriers and ocean currents. *Evolution* **53**, 806–817. (doi:10.1111/j.1558-5646.1999.tb05374.x)

49.	Shulman MJ. 2020 Echinometra sea urchins on Caribbean coral reefs: Diel and lunar cycles of movement and feeding, densities, and morphology. *J. Exp. Mar. Biol. Ecol.* **530–531**, 151430. (doi:10.1016/j.jembe.2020.151430)

50.	Young MAL, Bellwood DR. 2011 Diel patterns in sea urchin activity and predation on sea urchins on the Great Barrier Reef. *Coral Reefs* **30**, 729. (doi:10.1007/s00338-011-0754-2)
